# Sex differences in prostaglandin biosynthesis in neutrophils during acute inflammation

**DOI:** 10.1038/s41598-017-03696-8

**Published:** 2017-06-19

**Authors:** Simona Pace, Antonietta Rossi, Verena Krauth, Friederike Dehm, Fabiana Troisi, Rossella Bilancia, Christina Weinigel, Silke Rummler, Oliver Werz, Lidia Sautebin

**Affiliations:** 1 Department of Pharmacy, School of Medicine, University of Naples Federico II, Naples, 80131 Italy; 2 Chair of Pharmaceutical/Medicinal Chemistry, Institute of Pharmacy, Friedrich-Schiller-University, Jena, D-07743 Germany; 3 Institute of Transfusion Medicine, University Hospital Jena, Jena, 07747 Germany

## Abstract

The severity and course of inflammatory processes differ between women and men, but the biochemical mechanisms underlying these sex differences are elusive. Prostaglandins (PG) and leukotrienes (LT) are lipid mediators linked to inflammation. We demonstrated superior LT biosynthesis in human neutrophils and monocytes, and in mouse macrophages from females, and we confirmed these sex differences *in vivo* where female mice produced more LTs during zymosan-induced peritonitis versus males. Here, we report sex differences in PG production in neutrophils during acute inflammation. In the late phase (4–8 hrs) of mouse zymosan-induced peritonitis and rat carrageenan-induced pleurisy, PG levels in males were higher versus females, seemingly due to higher PG production in infiltrated neutrophils. Accordingly, human neutrophils from males produced more PGE_2_ than cells from females. Increased PG biosynthesis in males was accompanied by elevated cyclooxygenase (COX)-2 expression connected to increased nuclear factor-kappa B activation, and was abolished when LT synthesis was pharmacologically blocked, suggesting that elevated PG production in males might be caused by increased COX-2 expression and by shunting phenomena due to suppressed LT formation. Conclusively, our data reveal that the biosynthesis of pro-inflammatory PGs and LTs is conversely regulated by sex with consequences for the inflammatory response.

## Introduction

Sex has emerged as contributing factor in the incidence and progression of diseases associated with the immune system, in particular inflammation, with implications for outcomes and therapies. Most striking sex differences have been observed in asthma and autoimmune diseases (autoimmune thyroid diseases, scleroderma, rheumatoid arthritis etc)^1^, a spectrum of pathologies in which the patient population is prevalently female. On the other hand, other innate immune disorders such as sepsis^2^ and post-surgery infections as well as gout^3^ display a higher incidence and severity in males. The molecular and cellular basis underlying this sex dimorphism is still not completely elucidated and could present important implications for a sex-specific pharmacotherapy.

Sex differences in eicosanoid (leukotriene (LT) and prostaglandin (PG)) production may be responsible, at least in part, for the sex-dependent incidence of many diseases related to inflammation. In fact, we recently reported about a sex-dimorphism in LT biosynthesis (higher in female)^4–6^ which is of relevance in the light of the well-known sex-biased incidence of several immune diseases, providing a link to asthma pathology in humans^7^. In particular, androgens exert inhibitory effects on LT formation in human innate immune cells (isolated neutrophils or monocytes, and human whole blood) resulting in a substantial lower LT formation in male cells compared to females^4, 5^. We recently confirmed these sex differences also *in vivo*, making use of a well-established model of acute inflammation, the zymosan-induced peritonitis in mice^6^. In fact, LT biosynthesis as well as the inflammatory response were significantly greater in the inflamed peritoneum of female versus male mice. On the cellular level, differential 5-lipoxygenase (LO) subcellular compartmentalization in human leukocytes and in murine peritoneal macrophages (PMs) from males and females might be the basis for the observed differences^4–6^.

LTs and PGs are locally acting bioactive lipid mediators derived from arachidonic acid (AA) produced by 5-LO and cyclooxygenase (COX) as key enzymes, respectively. They are markedly biosynthesized by monocytes/macrophages, neutrophils, and mast cells and regulate a diverse set of homeostatic and inflammatory processes^8^ linked to numerous sex-dependent diseases. PGs are formed by the action of COX (COX-1 and COX-2) enzymes in a two-step conversion of AA. First, COXs convert AA to a cyclic endoperoxide (PGG_2_) and incorporate a 15-hydroperoxy group. This hydroperoxy group of PGG_2_ is reduced to a hydroxy moiety yielding PGH_2_ that is subsequently converted to the corresponding PGs by specific PG synthases, the nature of which is determined by the enzyme content of the respective cell. COX-1 is constitutively expressed in most cells, thus regarded as a housekeeping protein. On the other hand, the expression of COX-2 is inducible and remains undetectable in most mammalian tissues under basal conditions. Exposure of several types of cells including fibroblasts, neutrophils and monocytes to bacterial endotoxins, cytokines, and hormones induces activation of mitogen-activated protein kinases (MAPK) and nuclear factor-kappa B (NF-κB) which, in turn, induce COX-2 expression and PG production^9^. Inhibiting the formation of PGs by aspirin and other non-steroidal anti-inflammatory drugs during inflammation remains a classic and prevailing strategy to alleviate pain, swelling and fever. A sex-dependent efficacy of aspirin has been underlined in several randomized trials^10^, suggesting differential production or signalling of PGs in males and females. Moreover, as interrelations and crosstalk between the two branches of AA transformation (COX/PGs and 5-LO/LTs) exist^11^, imbalances in LT biosynthesis between genders may translate into differential PG formation.

Here, we show sex differences in PG production in neutrophils during acute inflammation using two different *in vivo* experimental models, that are, mouse zymosan-induced peritonitis and rat carrageenan-induced pleurisy, as well as in human neutrophils *in vitro*. Higher PG levels in males are seemingly caused by (i) increased COX-2 expression connected to elevated NF-κB activation versus females and (ii) by AA shunting phenomena due to lower LT production in males.

## Results

### Sex differences in PG biosynthesis during zymosan-induced peritonitis in mice

**Figure 1:**
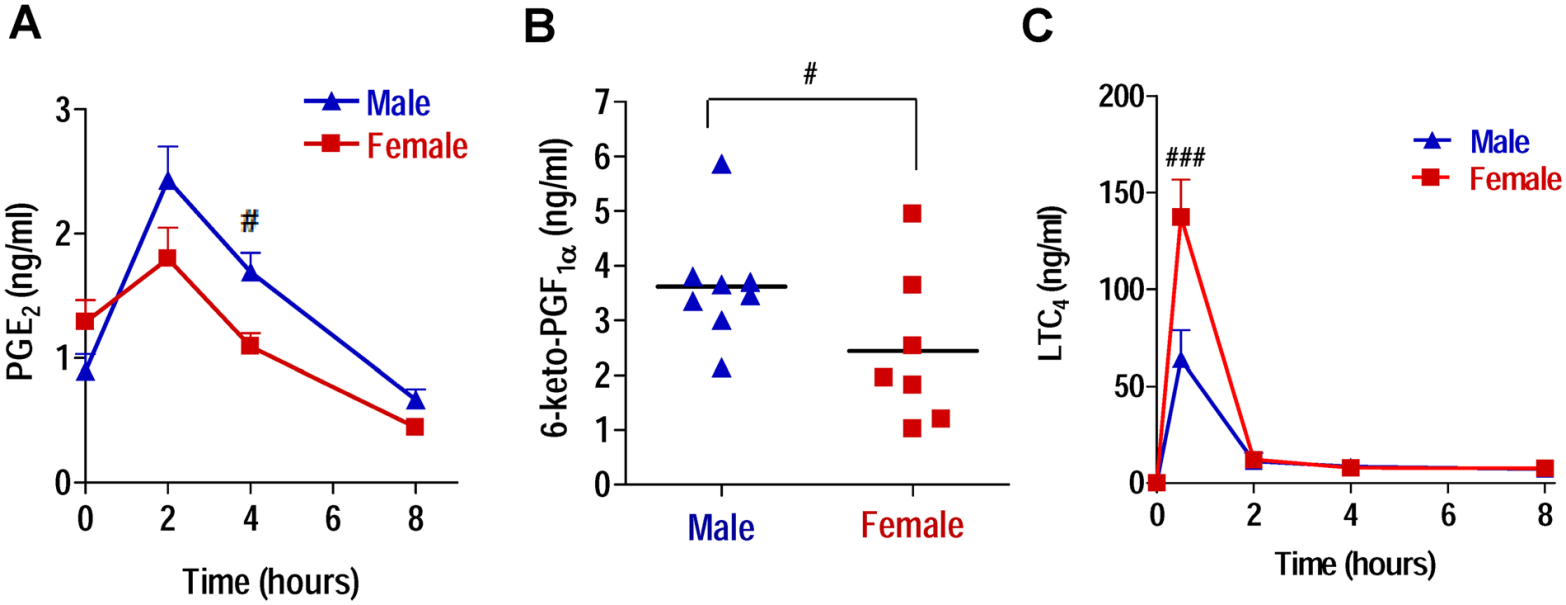
PGE_2_, 6-keto-PGF_1α_ and LTC_4_ peritoneal levels in male and female mice after zymosan-induced peritonitis. Mice were treated with zymosan (*i*.*p*.), sacrificed at the indicated time points and (**A**) PGE_2_ and (**C**) LTC_4_ were measured in peritoneal exudates from male and female mice, and (**B**) 6-keto-PGF_1α_ was measured in peritoneal exudates 4 hrs after zymosan injection. Time zero (T0) corresponded to untreated mice. Values represent means ± S.E.M; *n* = 10 mice/T0; *n* = 10 mice/2 hrs; *n* = 10 mice/4 hrs; *n* = 6 mice/8 hrs. ^###^p < 0.001 and ^#^p < 0.05, male vs female mice; two-way ANOVA plus Bonferroni (**A** and **C**) and two-tailed Student’s t test (**B**).

We have previously demonstrated that LT formation in zymosan-induced peritonitis in mice is higher in females compared to males, seemingly due to a divergent subcellular localization of 5-LO in LT-producing peritoneal macrophages^6^. Here, we investigated the temporal PG biosynthesis after intraperitoneal zymosan injection in male and female mice. Both sexes showed a similar time-course in the production of PGE_2_ with a peak after 2 hrs and continuous decrease until 8 hrs. Intriguingly, in male mice significant higher levels of PGE_2_ in the later phase of inflammation (p < 0.05 at 4 hrs; Fig. 1A) were evident in the peritoneal exudates after zymosan challenge, where the inflammatory cell population was mostly composed of neutrophils^6, 12, 13^. Moreover, in the exudates of male mice after 4 hrs, also higher levels of other PGs, such as 6-keto-PGF_1α_, the stable metabolite of PGI_2_, were evident as compared with females (p < 0.05, Fig. 1B). In contrast, the levels of LTC_4_ that peak after 15 min upon zymosan injection were higher in exudates from female versus male mice (Fig. 1C), which is in agreement with our previous report^6^.

### Sex differences in eicosanoid biosynthesis in carrageenan-induced pleurisy in rats

**Figure 2:**
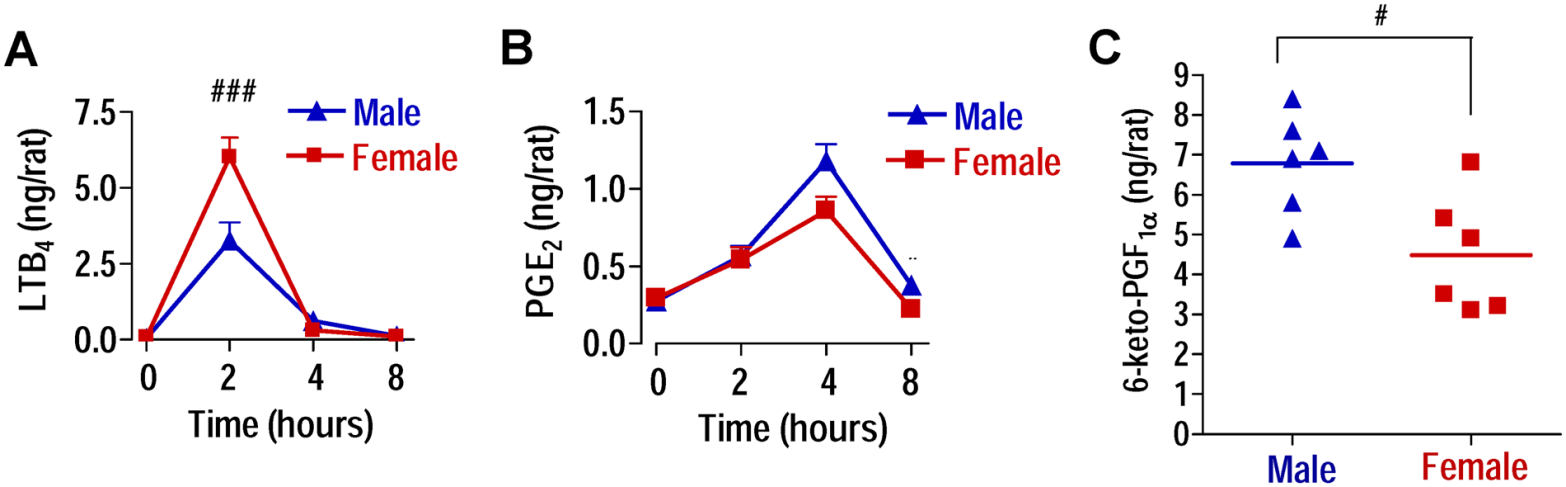
Sex differences in eicosanoid biosynthesis in carrageenan-induced pleurisy in rats. Rats were treated with carrageenan (intrathoracic injection), sacrificed at the given time points, and (**A**) LTB_4_ and (**B**) PGE_2_ in the thoracic exudates were measured by EIA and RIA, respectively. (**C**) 6-keto-PGF_1α_ levels in thoracic exudates were measured 4 hrs after carrageenan injection by EIA. Time zero (T0) corresponds to untreated rats. Values represent means ± S.E.M; *n* = 6 rats/T0; *n* = 13 rats/2 hrs; *n* = 6 rats/4 hrs; *n* = 6 rats/8 hrs; ^#^p < 0.05; ^###^p < 0.001, male vs female﻿;﻿ two-way ANOVA plus Bonferroni (**A** and **B**) and two-tailed Student’s t test (**C**).

Carrageenan-induced pleurisy in rats was chosen as another model of acute inflammation to investigate the sex-related regulation of eicosanoid biosynthesis. Two hrs after pleurisy induction, a peak of LTB_4_ was observed in both sexes, with a decrease at the later time points (4–8 hrs). In agreement with the data from the peritonitis model in mice^6^, higher levels of LTB_4_ were found in exudates of female versus male rats (p < 0.001, Fig. 2A) at the peak time (i.e., 2 hrs). The opposite was evident for PGE_2_ levels. Thus, a peak of PGE_2_ production was reached in both sexes 4 hrs after carrageenan injection and higher PGE_2_ levels were obvious in exudates of males, as observed in the murine peritonitis model. At 8 hrs, PGE_2_ levels dropped but the sex difference was still preserved (Fig. 2B). Moreover, a significant (p < 0.05) higher production of 6-keto-PGF_1α_ was found in male exudates 4 hrs after carrageenan injection (Fig. 2C), correlating to elevated PGE_2_ levels. Taken together, sex differences exist in PG biosynthesis *in vivo* during acute inflammation in two different animal models (mice vs. rats, zymosan vs. carrageenan, peritonitis vs. pleurisy) where PG levels were significantly higher in males versus females.

### Cyclooxygenase-2 expression and NF-κB activation in thoracic exudates differ between male and female rats

**Figure 3:**
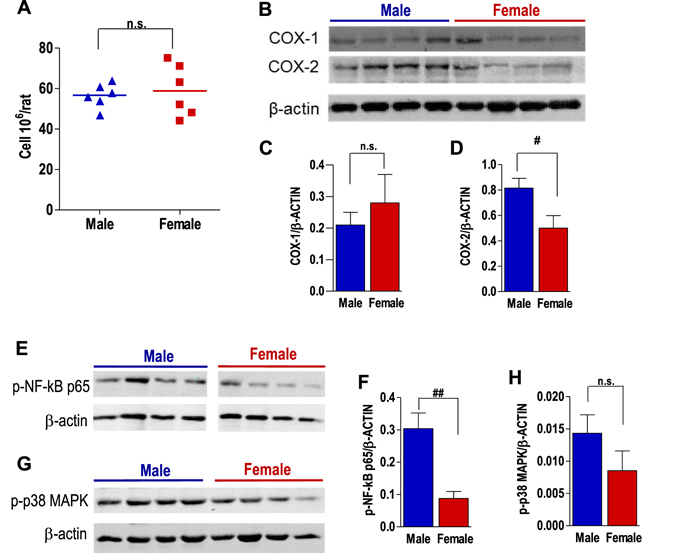
Cyclooxygenase expression and NF-κB activation in thoracic exudates differ between male and female cells. Rats were treated with carrageenan (intra thoracic injection) and sacrificed after 4 hrs. (**A**) Numbers of infiltrated cells (10^6^/rat) were measured in a Burker chamber using light microscopy. (**B**) Analysis of COX-1/2, (**E**) phospho-NF-κB p65 and (**G**) phospho-p38 MAPK by Western blot in total cell lysates of thoracic exudates from male and female carrageenan-treated rats. Densitometric analysis of (**C**) COX-1, (**D**) COX-2, (**F**) phospho-NF-κB p65 and (**H**) phospho-p38 MAPK protein normalized to β-actin expression. Values represents means ± S.E.M., *n* = 4 rats. ^#^p < 0.05; ^##^p < 0.01, male vs female; two-tailed Student’s t test.

Next, we investigated if the higher production of PGE_2_ in male animals during the inflammatory response was due to a higher number of PGE_2_-producing cells that infiltrate and mainly consist of neutrophils^14^. However, 4 hrs after pleurisy induction by carrageenan in rats, the cell number was not different between sexes (Fig. 3A). COX-1 and -2 play key roles in the biosynthesis of PGs, and the capacity of cells to produce PGs strongly depends on the amounts of COX enzymes expressed. Based on the sex difference in PG biosynthesis 4 hrs after pleurisy induction in rats, we analyzed the expression of COX-1 and -2 in the infiltrated cells by Western Blot (WB). No significant sex difference was observed for COX-1 expression, whereas COX-2 protein levels were higher in male cells (Fig. 3B,D), with respect to female counterparts. Interestingly, the sex difference in COX-2 protein expression was coupled to a significant higher activation status of NF-κB in male cells, visualized by elevated phospho-NF-κB p65 levels (Fig. 3E and F). Moreover, activation of p38 MAPK was more pronounced in cells from carrageenan-treated male rats as compared to cells from female animals (Fig. 3G and H), although the differences did not reach statistical significance.

### Inhibition of 5-LO product formation abolishes the sex differences in PG formation in the thoracic exudates of carrageenan-treated rats

**Figure 4:**
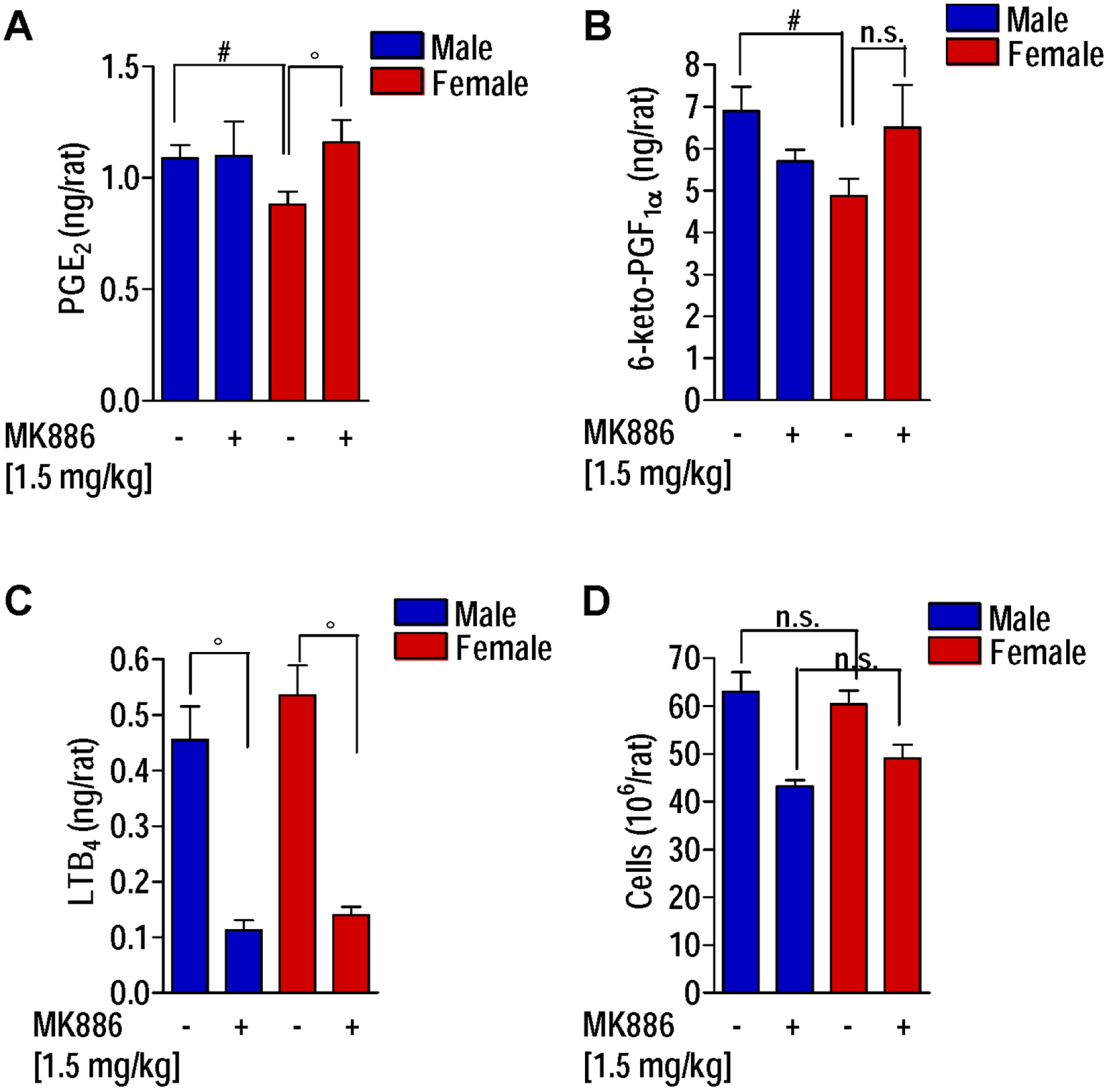
Inhibition of 5-LO product formation by MK886 abolishes the sex differences in PG formation in the thoracic exudates of carrageenan-treated rats. Rats were pre-treated with MK886 (1.5 mg/kg, i.p.) or vehicle (DMSO 2%, i.p.) 30 min prior to carrageenan intrathoracic injection and sacrificed after 4 hrs. (**A**) PGE_2_, (**B**) 6-keto-PGF_1α_, and (**C**) LTB_4_ levels in the exudates. (**D**) Number of infiltrated cells. Values represent means ± S.E.M; *n* = 6 rats; ^#^p < 0.05, male *vs* female; ^°^p < 0.05, female control rats vs female rats treated with MK886; two-tailed Student’s t test.

Based on the fact that LTB_4_ levels in the early phase (2 hrs) of the inflammatory response were lower in male versus female rats, we hypothesized that shunting phenomena from LTs to PGs might contribute to the opposite PG levels. Thus, we attempted to block 5-LO product formation by a pharmacological inhibitor (i.e. MK886)^15^ in order to investigate if the sex difference in PG biosynthesis at 4 hrs could be abolished. Rats were pre-treated with MK886 (1.5 mg/kg, i.p.) 30 min prior pleurisy induction, sacrificed after 4 hrs, and PGE_2_ levels were analyzed in the exudates. MK886 administration significantly (p < 0.05) abolished the sex bias in PGE_2_ (Fig. 4A) as well as in 6-keto-PGF_1α_ production (Fig. 4B). Furthermore, pre-treatment of rats with 1.5 mg/kg MK886 efficiently suppressed LTB_4_ levels by 75 and 74% in exudates of male and female animals, respectively (Fig. 4C). In agreement with this finding, MK886 treatment reduced the number of infiltrated cells into the thoracic cavity without significant differences between male and female animals (Fig. 4D).

### The capacity for PGE_2_ production in human neutrophils is sex-dependent

**Figure 5:**
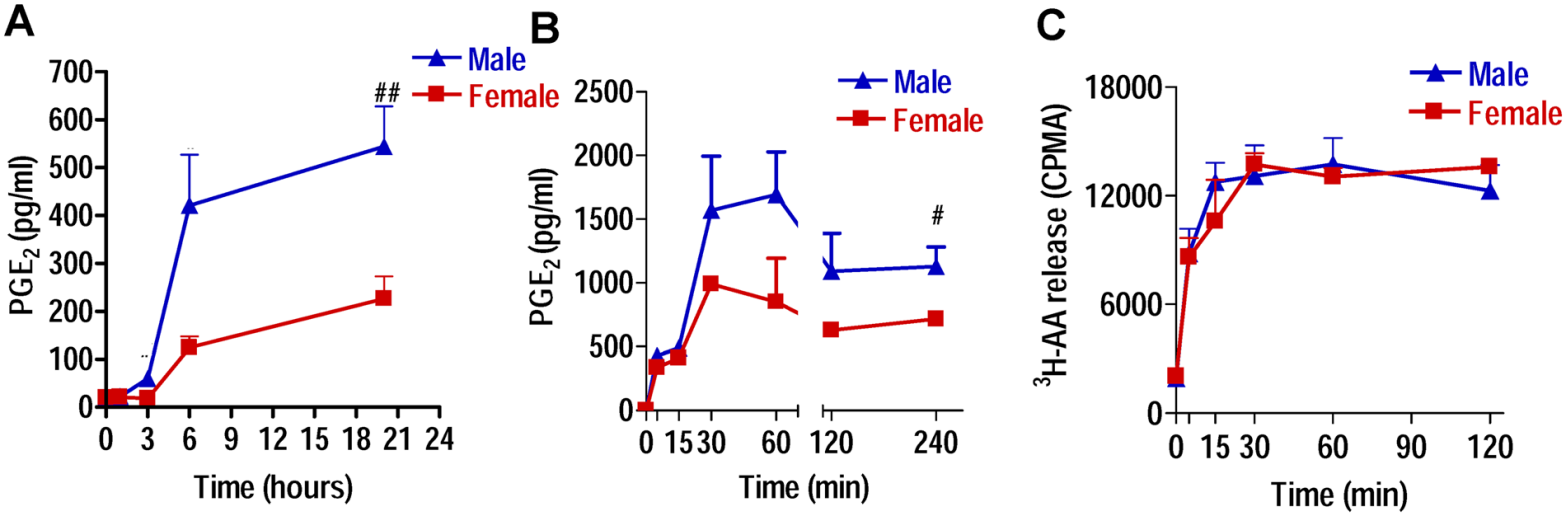
Capacity of human neutrophils for PGE_2_ production is sex-dependent. PGE_2_ production in human neutrophils from male and female donors after LPS or A23187 stimulation. Neutrophils from male and female donors were incubated with (**A**) LPS (1 µg/ml) for 0, 0.5, 3 and 20 hrs, or (**B**) with 0.5 µM A23187 for 0, 5, 15, 30, 60, 120, and 240 min. The reactions were stopped on ice, and PGE_2_ levels were measured by ELISA. (**C**) After selected time points (0, 5, 15, 30, 60, 120 min) upon stimulation with A23187 (0.5 µM), free ^3^H-AA content in the supernatant of ^3^H-AA-pre-labelled neutrophils was evaluated by scintillation counting. Values represents means ± S.E.M of *n* = *3*–*6* experiments each in duplicate. ^*#*^p < 0.05, ^##^p < 0.01, male vs female cells; two-way ANOVA plus Bonferroni.

Neutrophils are immune cells involved in the inflammatory reaction, recruited by chemoattractants (i.e. LTB_4_)^16^ that are produced by resident cells^17, 18^. To investigate if the sex difference in PGE_2_ biosynthesis exists also in humans, neutrophils from blood of female and male donors were freshly isolated and immediately stimulated for PGE_2_ production by lipopolysaccharide (LPS), a receptor-coupled stimulus, or by A23187 that causes cell activation by substantial mobilization of intracellular Ca^2+^ in a receptor-independent manner. Formation of PGE_2_ upon stimulation with LPS (1 µg/ml), which is strongly upregulated over the time course of 20 hrs, was much more pronounced for neutrophils derived from male versus cells from female donors (Fig. 5A). Also in response to A23187 (0.5 µM), a trend of higher PGE_2_ biosynthesis from male cells was evident starting at 30 min post stimulation and reaching significance (p < 0.05) at 4 hrs (Fig. 5B). Increased availability of AA as substrate in male cells may account for higher PGE_2_ synthesis. However, in agreement with our previous findings^4^, we observed no differences in the release of AA upon neutrophil stimulation with 0.5 µM A23187 between cells from male and female donors (Fig. 5C).

### Cyclooxygenase-2 expression is higher in male human neutrophils

**Figure 6:**
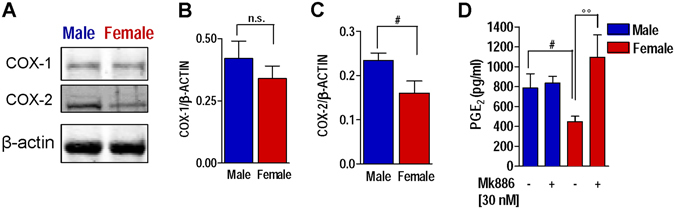
COX-2 expression is higher in neutrophils from male versus female donors; effects of 5-LO pathway inhibition on PGE_2_ production. (**A**–**C**) Western blot analysis of total cell lysates of neutrophils from male and female donors. (**A**) COX-1 and -2 protein expression. Densitometric analysis of (**B**) COX-1 and (**C**) COX-2 protein normalized to β-actin expression. Western blots are representatives of 11 independent experiments. Values represents means ± S.E.M of densitometric analyses of COX-1/-2 protein bands, normalized to β-actin. ^*#*^p < 0.05, male vs female cells. (**D**) PGE_2_ production in human neutrophils from male and female donors after pre-treatment with MK886 (30 nM, 15 min) upon A23187 stimulation (0.5 µM, 4 hrs). Values represents means ± S.E.M of *n* = *5* experiments, each in duplicate. ^#^p < 0.05, male vs female; ^°°^p < 0.01, female cells with MK886 vs female control cells; two-tailed Student’s t test.

Next, we addressed if COX expression in human neutrophils is affected by the sex. Analysis of COX protein levels (normalized to β-actin) in freshly isolated neutrophils from male and female donors by Western blot revealed no sex difference in the expression of COX-1 (Fig. 6A,B), while a significant (p < 0.05) higher expression of COX-2 in male cells was observed (Fig. 6A,C). This suggests that elevated PGE_2_ formation in male neutrophils might be connected to higher amounts of COX-2.

### The sex difference in PGE_2_ production in human neutrophils is abolished by inhibition of 5-LO product synthesis

Human neutrophils from females produce significantly higher levels of LTs from AA than cells from males^4^, implying that in male neutrophils more AA might be available as substrate for COX to produce PGs. In line with the data obtained from the carrageenan-induced pleurisy in rats, pre-treatment of neutrophils with MK886 (30 nM, 15 min) to block 5-LO product formation did not affect PGE_2_ formation in A23187-stimulated male neutrophils, while it significantly (p < 0.01) increased PGE_2_ synthesis in female cells. Thus, suppression of 5-LO product biosynthesis abolishes the sex difference in PGE_2_ formation also in human neutrophils (Fig. 6D).

## Discussion

A sex bias in inflammatory and immune diseases is clearly evident^1^, but the underlying biochemical or molecular mechanisms remain unclear. Here, we report about sex differences in PG production in human neutrophils *in vitro* and in rats and mice during acute inflammation *in vivo*. Our data suggest that the higher PG levels in neutrophils of males is due to AA substrate shunting phenomena because of lower LT production, but is also governed by higher amounts of COX-2 protein, as compared to neutrophils from females. The two major branches of biosynthetic pathways of pro-inflammatory lipid mediators produced from AA are the COX-mediated cascade leading to PGs and the 5-LO-mediated cascade yielding LTs^19^. Sex differences in the regulation of 5-LO in human leukocytes with consequences for LT formation were reported by us before^4, 5^, and we recently confirmed sex-biased LT biosynthesis in murine zymosan-induced peritonitis *in vivo* ^6^, a well-recognized model of acute inflammation^12^. Thus, we observed more pronounced LT formation and related inflammatory reactions (i.e., neutrophil infiltration and vascular permeability) in female mice as compared to male animals. The cells responsible for the observed sex difference were resident PMs, occupying the peritoneal cavity under normal physiological conditions, which are the first cells to respond to inflammatory stimuli. In analogy to human neutrophils^4^, the subcellular localization of 5-LO in female and male murine PMs differs, and the limited amount of mobile 5-LO in male PMs is seemingly responsible for lower LT biosynthesis^6^.

The results of the present report confirm higher LT formation in females but also reveal a sex-biased production of PG, in particular by neutrophils. However, while LT formation was higher in female cells and animals^4–6^, PG formation was higher in males. Our data are consistent with several observations made *in vitro* and *in vivo* studies observing a sex dimorphism in PG production, with higher levels in males or in ovariectomized female subjects^20–23^.

A time-course study of PGE_2_ biosynthesis in zymosan-induced peritonitis revealed higher PGE_2_ levels in exudates from male mice compared to female animals, 4 hrs or later after zymosan injection. Although PGE_2_ levels peaked at 2 hrs, the sex difference was significant only at 4 and 8 hrs, but not earlier. It should be noted that 4 hrs after peritonitis induction the cellular population of the peritoneal cavity is mostly composed of neutrophils as shown previously by us^6^ and others^12, 13^. Thus, we suggest that the sex difference is attributable to neutrophils, rather than to PMs that seem to equally generate PGs in the early phase (0–2 hrs) independently of the sex^6^. Surprisingly, despite the higher PGE_2_ production in males, the number of infiltrating cells were lower at 4 and 8 hrs as compared to female animals. This might be due to reduced levels of LTB_4_ in males^6^ that acts as potent chemotactic factor for neutrophils^18^, and based on its greater abundance in female mice may recruit neutrophils more efficiently. To confirm the general validity of sex differences in PG biosynthesis, we chose a different animal model of acute inflammation (i.e. pleurisy) with a different stimulus (i.e. carrageenan) and different species (i.e. rat) to support sex-dependent production of eicosanoids as general and model-independent phenomenon. Carrageenan-induced pleurisy in rats represents one of the most commonly used models to investigate eicosanoid biosynthesis and signaling during acute inflammation^19, 24–26^. In agreement with the results from the zymosan-induced peritonitis, significant higher LTB_4_ levels in thoracic exudates of female rats were evident 2 hrs after carrageenan-injection, while superior PGE_2_ levels were found in thoracic exudates of male animals at later time points (4–8 hrs). These data highlight the converse transformation of AA to PGs (higher in males) and LTs (higher in females), and suggest that sex differences in PGE_2_ production in these experimental models are most likely related to neutrophils. During acute inflammation, the mobilization and recruitment of blood leukocytes into the tissue are mediated by several factors^27^. Among them, LTB_4_ through the BLT1 receptor might be the major chemoattractant molecule responsible for neutrophil infiltration^18^. Since the LTB_4_ levels were higher in the thoracic exudates of female rats, we hypothesized that this would cause an increased neutrophil infiltration in the cavity of female animals. Notably, however, the number of infiltrated neutrophils did not differ between males and females at 4 hrs after carrageenan, and MK886 at 1.5 mg/kg that strongly repressed LTB_4_ formation and caused significant inhibition of cell infiltration in both sexes.

For PG production, AA is converted in two steps by the action of COX enzymes that catalyze the transformation of AA into the endoperoxide PGG_2_ containing a 15-hydroperoxy moiety. Reduction of the hydroperoxy group to a hydroxyl function then leads to PGH_2_ that is further converted by specific PG synthases to the respective bioactive PGs, including PGE_2_, PGF_2_, PGD_2_, PGI_2_ and thromboxane(s), depending on the tissue-selective expression of the PG synthases^28^. Since besides PGE_2_ also the 6-keto-PGF_1α_ (a stable metabolite and marker of instable PGI_2_) was consistently higher in males, these data support that upstream COX enzymes might be affected by the sex rather than mPGES-1, the terminal enzyme in pro-inflammatory PGE_2_ biosynthesis^29^. Nevertheless, sex differences related to mPGES-1 were found in spontaneously hypertensive rats, where female rats had enhanced mPGES-1 protein expression in the renal inner medulla and greater COX-2 expression in the outer medulla versus males^21^. In our hands, COX-2 protein, but not COX-1, was more abundant in cells from thoracic exudates of male rats and in human neutrophils from males, and such dominance of COX-2 in males is in agreement with observations by others. Thus, lower COX-2 expression and activity has been noted in the *macula densa* of female rats compared to males, contributing to the major protection of female to the blood pressure increment and renal damage^30^. Moreover, the inferior susceptibility to traumatic brain injury of male versus female rats has been related to a robust higher expression of COX-2 in the brain of male rats^31^. In addition, long-term testosterone treatment augmented COX-2 levels in male rat brain blood vessels, whereas treatment of male rats with 17β-estradiol significantly impaired cerebrovascular COX-2 levels after an inflammatory stimulus^21^.

Expression of COX-2 is regulated by several transcription factors including NF-kB^9^, whose activation in thoracic cells from carrageenan-treated rats was sex-dependent in our present study. In fact, phospho-NF-κB p65 levels in male rats were significantly higher with respect to female animals. Our data are in line with previously reported sex differences in cerebrovascular pathophysiology that were due to activation of the NF-κB-mediated COX-2 pathway by the androgen 5α-DHT that results in a state of vascular inflammation^32^.

Our data reveal that activation of human neutrophils by LPS or by A23187 leads to significantly higher PGE_2_ levels in cells from males versus female counterparts. We showed before that stimulation of human neutrophils with LPS plus fMLP or with A23187 caused higher LTB_4_ production in female cells as compared to males^4^. Although A23187 preferentially activates the 5-LO pathway via receptor-independent, massive elevation of intracellular Ca^2+^, we believe that it represents a suitable stimulus to investigate the sex-regulation of eicosanoid biosynthesis, considering the fact that other stimuli (e.g., LPS) act through receptors that are strongly modulated by sex as well^33, 34^. We hypothesized that the blockade of 5-LO product formation by using MK886 would redirect AA conversion by COX enzymes. In fact, the sex difference in PGE_2_ formation in human neutrophils *in vitro* as well as in carrageenan-treated rats *in vivo* was abolished by interruption of 5-LO product formation using MK886 that significantly increased PGE_2_ in females without any alterations in males. In parallel, MK886 strongly reduced LTB_4_ levels in both test systems. On the other hand, blockade of PGE_2_ production may have the converse phenotype, however, the COX inhibitor indomethacin did not increase LTB_4_ levels in male rats during carrageenan-induced pleurisy^35^.

Taken together, we showed that male mice and rats produce higher levels of PGs in various acute models of inflammation *in vivo* under conditions where LT production is elevated in female animals at the sites of injury. Neutrophils are abundant innate immune cells in the human body taking part of the first line of defense against host injury, and are considered to be a major source of LTs^33^. Our findings imply that neutrophils from male subjects have higher capacities to produce PG seemingly due to elevated COX-2 expression and AA substrate availability. These sex differences are of relevance for PG-related functions and pathophysiology, supported also by experimental observations reported by others, and might help to explain, at least in part, the sex dimorphism in innate immune disorders such as sepsis^2^ and post-surgery infections as well as gout^3^.

## Material and Methods

### Materials

Enzyme immunoassay (EIA) kits were from Cayman Chemical Company (BertinPharma, Montigny Le Bretonneux, France) or from Biotrend (Cologne, Germany). ^3^H-labelled PGE_2_ and ^3^H-labelled AA were from PerkinElmer Life Sciences (Milan, Italy and Germany). Unless otherwise stated, all other reagents and compounds were obtained from Sigma-Aldrich (Milan, Italy).

### Animals

The animal studies are reported in accordance with the ARRIVE guidelines for reporting animal research^36^. Age-matched male and female CD-1 mice (8–9 weeks old, 26–40 g Charles River, Calco, Italy) and Wistar male and female rats (200–300 g, Harlan, Milan, Italy) were housed in a controlled environment (21 ± 2 °C) and provided with standard rodent chow and water. All animals were allowed to acclimate for four days prior to experiments and were subjected to 12 h light–12 h dark schedule. Experiments were conducted during the light phase. The experimental protocols were approved by the Animal Care Committee of the University of Naples Federico II, in compliance with Italian regulations on protection of animals used for experimental and other scientific purpose (Ministerial Decree 26/2014) as well as with the European Economic Community regulations (Official Journal of E.C. L358/1 12/18/1986).

### Induction of peritonitis in mice

Peritonitis was induced in mice as previously described^6^. In brief, a solution of 2 mg/ml of zymosan A (boiled and washed) was injected intraperitoneally (i.p., 0.5 ml) and at selected time points (0–2–4–8 hrs), mice were sacrificed in a saturated atmosphere with CO_2_. Peritoneal exudates were collected by washing the cavity with 2 ml of phosphate-buffered saline (PBS) and then centrifuged at 20,000 × *g* for 20 min at 4 °C and supernatants frozen at −80 °C for measurements of eicosanoids. PGE_2_ was evaluated by radioimmunoassay (RIA), 6-keto-PGF_1α_ and LTC_4_ by EIA (Cayman chemicals BertinPharma, Montigny Le Bretonneux, France), according to manufacturer’s protocol. Results are expressed as ng/ml.

### Induction of pleurisy in rats

Rats were anesthetized with 4% enflurane mixed with 0.5 l/min O_2_, 0.5 l/min N_2_O and submitted to a skin incision at the level of the left sixth intercostal space. The underlying muscle was dissected and 1% (w/v) λ-carrageenan type IV (0.2 ml) was injected into the thoracic cavity. The skin incision was closed with a suture and the animals were allowed to recover. At selected time points (0–2–4–8 hrs) after carrageenan injection, animals were sacrificed by CO_2_ inhalation. Thoracic exudate was collected by lavage of the cavity with 2 ml of saline solution, after centrifugation (800 × *g* for 10 min), the leukocyte number was determined by light microscopy using a Bürker chamber. The cells as well as the supernatants were frozen at −80 °C for WB analysis and eicosanoid measurement, respectively. In one set of experiments, rats were pre-treated with 1.5 mg/kg MK886 (Cayman Chemical, Bertin Pharma, Montigny Le Bretonneux, France) or vehicle (2% DMSO in saline) 30 min prior to pleurisy induction. Animals were then sacrificed 4 hrs after carrageenan injection. The amount of PGE_2_, LTB_4_ and 6-keto-PGF_1α_ in the supernatant of centrifuged exudates was measured by RIA and by EIA, respectively. Results are expressed as the total amount of eicosanoid measured in the thoracic exudate of one rat (nanograms per rat).

### Isolation and stimulation of human neutrophils

Leukocyte concentrates, prepared from freshly withdrawn peripheral blood of healthy adult human donors who had not taken any anti-inflammatory drugs for the last 10 days were obtained from the Institute of Transfusion Medicine at the University Hospital Jena, Germany. Informed consent was obtained from all subjects. The experimental protocol was approved by the local ethical committee at the University Hospital Jena. All methods were performed in accordance with the relevant guidelines and regulations. Neutrophils were isolated as previously described^4, 37^. In brief, neutrophils were obtained from leukocyte concentrates by a multi-step procedure: (1) dextran sedimentation; (2) centrifugation on Nycoprep (872 × *g*, 10 min); (3) hypotonic lysis of erythrocytes. Finally, cells were suspended in ice-cold PBS containing 0.1% glucose (PG buffer) and counted by Vi-CELL™ XR. For PG production, 5 × 10^6^ neutrophils, from female and male donors, were resuspended in PG buffer containing 1 mM CaCl_2_ (PGC buffer) and stimulated with 1 µg/ml LPS for 0, 0.5, 3 and 20 hrs or with 0.5 μM A23187 for 0, 5, 15, 30, 60, 120, or 240 min. In one set of experiments, freshly isolated neutrophils from male and female donors were pre-treated with 30 nM MK886 or DMSO as vehicle (15 min., 37 °C), and then stimulated with 0.5 μM A23187 for 4 hrs. The reaction was stopped on ice and samples were centrifuged (12,000 × *g*, 5 min, 4 °C), PGE_2_ levels in the supernatants were measured with ELISA kit (Biotrend, Cologne, Germany).

### Total protein extraction and Western blot analysis

Protein analysis of COX-1/2, phospho-NF-κB p65, phospho-p38 MAPK and β-actin by Western blot was performed in whole cell lysates. Cells in the thoracic exudates were collected 4 hrs after carrageenan administration and then immediately lysed in a buffer for protein extraction, mixed with sodium dodecyl sulphate (SDS) loading gel buffer and analysed by Western blot according to ref. 6 on a 10% SDS–polyacrylamide gel. The membranes were incubated overnight with rabbit monoclonal antibody anti-COX-2 (1:500, BD Transduction Laboratories, Aurogene, Rome, Italy), mouse monoclonal antibody anti-COX-1 (1:1000, Cell Signaling, Aurogene, Rome, Italy), rabbit monoclonal antibody anti-phospho-NF-kB p65 (Ser536) (1:1000, Cell Signalling Technology, Inc., Germany), rabbit polyclonal antibody anti-phospho-p38 MAPK (Thr180/Thr182) (1:1000, Cell Signalling Technology, Inc., Germany) or β-actin (1:2000, Santa Cruz Biotechnology, Aurogene, Rome, Italy). Membranes were washed six times with 0.1% PBS-Tween and were incubated for 1.5 hrs at room temperature with horseradish peroxidase-conjugated anti-rabbit and anti-mouse secondary antibodies diluted 1:10,000 in 0.1% PBS-Tween containing 5% non-fat dry milk. Protein bands were detected by an enhanced chemiluminescence system (Amersham Pharmacia, Aurogene, Rome, Italy). Densitometric analysis was performed by using the Fluor S quantitative imaging system.

Human neutrophils from female and male donors were isolated and rapidly processed for protein extraction as previously described^4^. Briefly, 3 × 10^7^ cells/ml PBS plus 1 mM EDTA were sonicated (3 × 5 sec, 4 °C) and centrifuged (12,000 × *g*, 15 min, 4 °C). Aliquots of the resulting supernatants were mixed 1:1 with ice-cold 2× SDS/PAGE sample loading buffer (SDS-b) and heated for 6 min at 95 °C. Samples were loaded (15 µl) and electrophoresed on a 10% SDS–polyacrylamide gel, and transferred to nitrocellulose membranes. After the membranes were incubated with primary antibodies (rabbit anti-COX-1, 1:1000, Cell signaling; rabbit anti-COX-2, 1:1000, Santa Cruz; mouse anti-β-actin, 1:1000; Santa Cruz) they were subsequently detected using IRDye 800CW-labeled anti-rabbit and/or anti-mouse antibodies (1:10,000 each). The immunoreactive bands were visualized using an Odyssey infrared imager (Li-Cor Biosciences, Lincoln, NE).

### Arachidonic acid release in human neutrophils

Arachidonic acid release in neutrophils from male and female donors was evaluated as reported previously^38^. Briefly, freshly isolated neutrophils (2 × 10^7^ cells/ml) were re-suspended in RPMI 1640 without additives, 0.5 µCi ^3^H-labelled AA/ml were added to the cell suspension and incubated for 2 hrs at 37 °C. Cells were washed twice (320 × *g*, 10 min, 4 °C) with incubation buffer (PBS, containing 0.1% glucose and 2 mg/ml fatty acid free BSA). Cells were adjusted to a cell number of 1 × 10^7^/0.5 ml and 1 mM CaCl_2_ was added to the incubation buffer. Cells were simulated with Ca^2+^-ionophore A23187 (0.5 µM) for 5 to 120 min, as indicated, at 37 °C. The reaction was stopped on ice and samples were centrifuged (500 × *g*, 10 min, 4 °C). Aliquots (300 µl) of the supernatants were combined with 2 ml Rotiszint® eco plus and assayed for radioactivity by scintillation counting (Micro Beta Trilux, Perkin Elmer, Waltham, MA).

### Statistical analysis

Data are expressed as mean ± standard error of the mean (S.E.M.) of *n* observations, were *n* represents the number of animals, or the number of experiments (*in vitro*) performed with cells from different donors in duplicates. Statistical evaluation was performed by two-tailed Student t-test for single comparisons or by two-way ANOVA using GraphPad InStat (Graphpad Software Inc., San Diego, CA) followed by a Bonferroni post-hoc test for multiple comparisons, respectively. P-values < 0.05 were considered as significant.
